# Patients with neuromyelitis optica spectrum disorder (NMOSD) are associated with adverse outcome after total hip arthroplasty: a matched case–control study

**DOI:** 10.1186/s13023-021-02005-x

**Published:** 2021-08-30

**Authors:** Xi Chen, Wenwei Qian, Guixing Qiu, Xisheng Weng, Jin Lin, Jin Jin, Shibai Zhu, Yiou Wang, Shanni Li

**Affiliations:** Department of Orthopedic Surgery, Peking Union Medical College Hospital, Peking Union Medical College, Chinese Academy of Medical Science, Beijing, China

**Keywords:** Neuromyelitis Optica Spectrum Disorders, Total Hip Arthroplasty

## Abstract

**Background:**

Neuromyelitis Optica Spectrum Disorders (NMOSD) are rare inflammatory diseases of the central nervous system that cause transverse myelitis and optic neuritis. Steroids are commonly administered in NMOSD patients. The use of steroids may lead to osteonecrosis, which makes some of the NMOSD patients candidate for total hip arthroplasty (THA). To date, the clinical outcome of THA in NMOSD patients have not been investigated.

**Aim:**

Investigate the patient reported outcome measures (PROM), radiographic outcome and complication in NMOSD patients after THA, compared with that of non-NMOSD patients.

**Methods:**

Patients from Jan. 2016 to October. 2020 were identified in our database. 12 NMOSD cases which met the inclusion criteria were matched to non-NMOSD cases in a ratio of 1:2 based on age, sex, Charlson Comorbidity Index (CCI) and surgical date. Relevant outcome were analyzed and compared between the two groups.

**Results:**

There was a significantly increased risk of dislocation in NMOSD patients. Post-operative HOOS score was similar between the two groups even though the pre-operative HOOS score is significantly higher in the non-NMOSD group. NMOSD patients had poor performance in EQ-5D and EQ-VAS. The cups were placed more anteverted in NMOSD cases (*P* = 0.01).

**Conclusion:**

There is a significantly increased risk of dislocation after THA in NMOSD patients. However, satisfactory improvement in functional outcome of the hip was achieved. Due to the natural process of NMOSD, rehabilitation and hip precaution should be patient-specific and time-specific.

## Article summary

Article focus:

1. Investigate the complications, patient reported outcome measures and radiographic outcomes in Neuromyelitis Optica Spectrum Disorders patients who received total hip arthroplasty.
2. Compare the clinical outcome and radiographic outcome of NMOSD patients to a case-matched control group.
3. Explore the potential causes for the inferior clinical outcome in NMOSD cases and provide clinical suggestion.

## Key messages

1. A significantly higher rate of dislocation was found in NMOSD cases comparing with controls. Recurrent attacks of limb weakness and spasticity might be the cause for the high dislocation rate.
2. Satisfactory functional improvement was achieved in both groups as measured by HOOS. NMOSD cases scored lower in overall life quality score as measured by EQ-5D and EQ-VAS
3. Hip precaution and rehabilitation should be patient-specific and time-specific in NMOSD patients.

## Strength/limitation

1. To date, the clinical outcomes of THA in NMOSD patients have not been reported in literatures.
2. The sample size in this study is small (12 NMOSD cases). However, considering the low incidence of NMOSD, all available cases were gathered from the center of rare disease to form this case series.
3. A matched case control study was conducted to eliminate bias caused by confounding factors. Another benefit of matching is to gain more efficiency in studies with a small sample size.

## Introduction

Neuromyelitis Optica Spectrum Disorders (NMOSD) are rare inflammatory diseases of the central nervous system which cause disorders by astrocyte injury and secondary demyelination. The prevalence of NMOSD was reported to be 0.5 to 10 per 100,000 individuals. NMOSD predominantly affect women at 10:1 with median onset age of 32–41 [1]. The classic clinical presentations of NMOSD are recurrent attacks of demyelination which causes transverse myelitis and optic neuritis [2, 3]. Patients typically suffer from severe loss of vision, bladder dysfunction, limb weakness, sensory loss and dystonia, with a typically relapsing course [4]. Although NMOSD can be further classified into subtypes based on autoantibody, the frequent relapse, characteristics of clinical manifestation and identification of serologic antibody against aquaporin 4-immunoglobulin G(AQP4) make NMOSD a distinct entity from other inflammatory diseases. Given the poor prognosis of the disease, life-long therapy consisting of immunosuppressant and corticosteroids are recommended by literatures [5]. Half of the patients have severe motor impairment and loss of vision within 5 years, and the mortality rate can be as high as 25% [6].

NMOSD patients is at risk of osteonecrosis and trauma. Steroids are commonly used in NMOSD patients, which predispose them to osteonecrosis. High dose of intravenous methylprednisolone is recommended as initial treatment of acute attacks. Continuous low dose of corticosteroids can be used as maintenance therapy [7]. NMOSD patients are at higher risk of developing osteonecrosis and secondary osteoarthritis due to use of corticosteroids. Limb weakness, spasticity, sensory loss and visual impairment can lead to increased risk of trauma. Due to trauma and osteonecrosis, it is likely that a proportion of NMOSD patients will eventually receive hip arthroplasty.

Limb weakness, spasticity, sensory loss and even loss of vision have posed challenges to rehabilitation after arthroplasty and predispose patients to complications and worse functional outcome. Previous research showed that patients with neuromuscular disorder are associated with adverse outcome following THA [8]. However, the functional outcome and complications of NMOSD patients who underwent total hip arthroplasty have not been investigated.

Therefore, a retrospective matched case–control study was conducted, which aims to:1. Investigate the PROM, radiographic outcome and complications in NMOSD patients after THA and compare with that of non-NMOSD patients. 2. Explore the potential causes for different clinical outcomes between the two groups, and discuss how surgical techniques and peri-operative managements should be modified in NMOSD-patients.

## Material and methods

### Data source

Approval from the ethics committee and consent from each included patient were obtained. The study was carried out using the database from National Rare Disease Center. Discharge diagnosis was based on International Classification of Disease, 10th revision (ICD-10), surgical procedure was based on ICD-9, surgical date, discharge date, demographic characteristics and other parameters were extracted.

### Study population

Patients who received elective total hip arthroplasty between January 2016 and September 2020 were identified. Patients were included in the cohort if they underwent elective primary total hip arthroplasty. 3936 patients received elective total hip arthroplasty. Patients diagnosed with NMOSD were identified using the ICD-10 diagnostic codes. Diagnosis were confirmed through consultation with neurologists. Patients without NMOSD and other neuromuscular diseases formed the control group. Each NMOSD patient was matched to non-NMOSD patients at a 1:2 ratio based on age, sex, CCI and surgical date. The detailed matching criteria included: 1. Each control patient should be the same gender as the corresponding NMOSD patient; 2. The age difference between each control patient and corresponding NMOSD patient should be within ± 5 years. 3. The difference of Charlson Comorbidity Index (CCI) between each control patient and corresponding NMOSD patient should be within ± 1. 4. The difference of surgical date (follow-up time) should be within ± 3 months between each control patient and each corresponding NMOSD patient. If more than 2 patients from the control group were matched to the corresponding NMOSD patient. The 2 patients with the least difference in follow-up time were selected as the control patients.

### Outcome variables

Patient reported outcome measurement included Hip Disability and Osteoarthritis Score Joint Replacement (HOOS JR) [9], EuroQol 5 Dimensions Questionnaire (EQ-5D), EuroQol Visual Analogue Scale (EQ-VAS) [10], Visual analogue scale (VAS) and self-reported range of motion(ROM) were recorded preoperatively and at last follow up to assess the functional outcome, overall health status and pain. Pre-operative and post-operative AP Pelvis X-Ray were acquired in all cases. Radiographic outcome were measured using Digital Imaging and Communication in Medicine(DICOM). Cup inclination and anteversion were measured as described by Lewwinek et al. [11]. Post-operative limb length discrepancy were measured based on the distance between inter-ischial line and the bilateral upper edge of the lesser trochanter. Post-operative complications and other parameters were recorded.

### Surgical procedure and peri-operative management

All patients received cementless prothesis. The acetabular components were Pinnacle (DePuy Orthopaedics, Warsaw, IN, USA) and R3 (Smith&Nephew,, TN, USA). The femoral components were Corail Stem (DePuy Orthopaedics, Warsaw, IN, USA), Trilock stem (DePuy Orthopaedics, Warsaw, IN, USA) and ML-Taper Stem (Smith&Nephew,, TN, USA). Ceramic on ceramic bearings were used in all cases. The surgeries were performed through posterior approach and direct anterior approach. Standard peri-operative treatment and patient education were applied. For NMOSD patients, a multidisciplinary team including neurologist, orthopedist and anesthesiologist assess the condition of the patients and decide if immunosuppressant, corticosteroids or any other treatment for NMOSD should be modified during the perioperative period.

### Statistical analysis

Statistical analysis were performed with SPSS version 25 (IBM, New York, USA) and GraphPad Prism version 8 (GraphPad Software, CA, USA). Continuous variables were recorded as means and standard errors. Categorical variables were recorded as counts and percentages. Student t-tests were used to compare continuous variables except for PROM. Categorical variable were compare with Fisher exact test. PROM scores were analyzed with t tests and analysis of covariance (ANCOVA). ANCOVA was used to adjust for difference in pre-operative PROM scores and to provide an unbiased estimate of differences of post-operative PROM scores between the two groups.

## Results

### Study population

14 NMOSD cases who underwent total hip arthroplasty were identified in our database. The follow-up time ranged from 6 to 46 months with average follow-up time of 26.50 months. 1 case was excluded because she had acute femoral neck fracture and underwent femoral head replacement. 1 case was lost in follow-up. 12 cases were eventually included in the NMOSD group. 24 cases were included in the non-NMOSD group. The demographic characteristics of study population were listed in Table 1. After matching with sex, age, CCI and surgical date, there was no statistically significant difference in terms of age, sex, CCI, BMI and follow-up time. All cases had at least 6 months of follow-up. The steroids used in both groups were prednisone, prednisolone and methylprednisolone. In the NMOSD group, the time between initial steroids treatment and surgery ranged from 1.5 to 17 years. The maximal dosage of initial steroids treatment ranged from 50 mg/day to 60 mg/day. The steroids dosage were reduced to 5 mg/day to 10 mg/day prior to surgery. In the control group, the time between initial steroids treatment and surgery ranged from 1 to 20 years. The maximal dosage of steroids use during treatment ranged from 40 mg/day to 80 mg/day. The dosage of steroids use were 2 mg/day to 10 mg/day prior to surgery. The specific information of steroids used in the both group were listed in Table2.

**Table 1:** Demographic characteristics of included cases

	NMOSD (n = 12)	non-NMOSD (n = 24)	*P* value
Age (years)	40.58 ± 7.72	39.21 ± 9.16	0.658
Sex			
Female	12/12	24/24	
BMI	23.03 ± 3.23	22.97 ± 2.88	0.96
CCI	2.17 ± 0.39	1.95 ± 0.62	0.30
Follow-up time(months)	24.70 ± 11.16	24.43 ± 11.54	0.95

^*^Data were displayed as means and standard deviation and rate

**Table 2:** Information of steroids use

	Time of steroids use	Type of steroids used	Maximal dosage	Dosage prior to surgery
NMOSD group	1 to 20 years	Prednisone, prednisolone, Methylprednisolone	40–80 mg/day	2–10 mg/day
Control group	1.5 to 17 years	Prednisone, prednisolone, methylprednisolone	50–60 mg/day	5–10 mg/day

In the NMOSD group, 5 cases had lower limb weakness pre-operatively and at last follow-up. 3 cases developed lower limb weakness post-operatively. 5 cases had spasticity pre-operatively and at last follow-up. 2 cases developed spasticity post-operatively. Overall, 66.7% (8/12) of the cases in the NMO group had post-operative limb weakness and spasticity at last follow up. The average recurrent attack times were 1.92 during the last five years.

### Patient report outcome measures

**Fig. 1:**
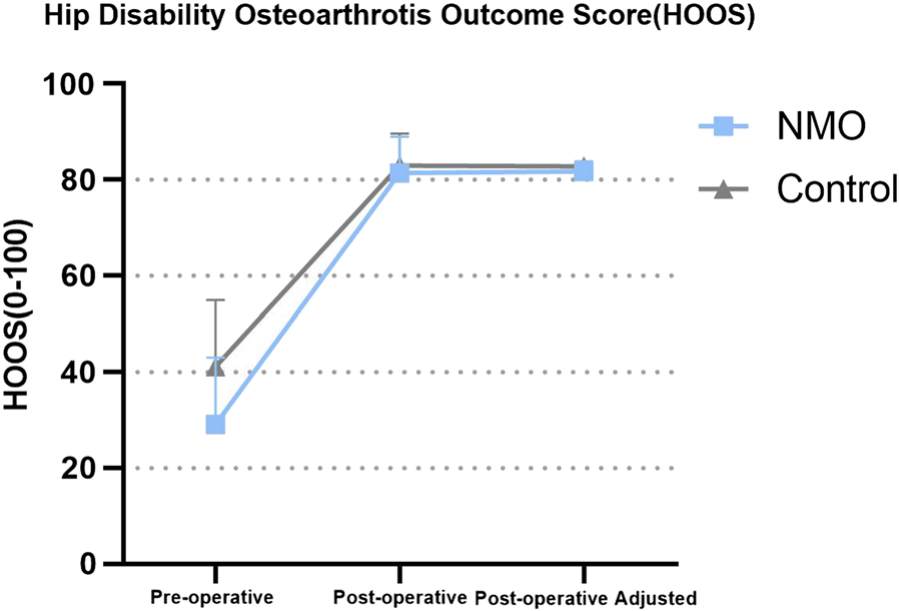
Hip disability osteoarthritis outcome (HOOS) score

**Fig. 2:**
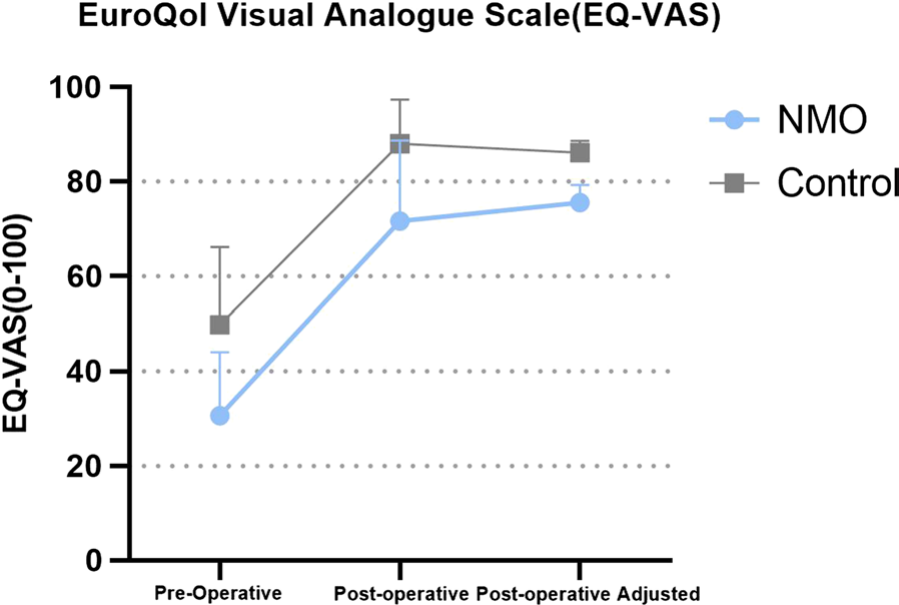
EuroQol Visual Analogue Scale (EQ-VAS)

Functional outcome was assessed by HOOS-JR in 5 dimensions: Symptoms, Pain, Activity in daily life(ADL), Sports and recreation(SR), Quality of life(QOL). Overall health status was assessed by EQ-5D and EQ-VAS. Pain was assessed by Visual Analogue Scale (VAS). The results were listed in Table 3. In terms of general HOOS score, the average pre-operative score was 29.06 ± 13.94 in NMOSD group and 41.12 ± 13.82 in non-NMOSD group. The post-operative HOOS score was 81.70 ± 2.16 in NMOSD group and 82.79 ± 1.84 in non-NMOSD group, the average difference was 52.34 ± 12.91 in NMOSD group and 41.82 ± 15.97 in non-NMOSD group. No significant difference in post-operative HOOS score was found after adjusting for pre-operative differences (Fig. 1). In terms of EQ-VAS, the pre-operatively average score was 30.58 ± 13.37 in NMOSD group and 49.79 ± 16.36 in the non-NMOSD group, the post-operatively average score 71.67 ± 16.97 in NMOSD group and 88.00 ± 9.30 in the non-NMOSD group, the average difference was 41.08 ± 15.65 in NMOSD group and 38.21 ± 15.70 in non-NOM group (Fig. 2). NMOSD group performed significantly worse in post-operative EQ-VAS (*P* = 0.035) and in post-operative EQ-Usual Activity. No significant difference was found in VAS score, which was recorded in Table 3.

**Table 3:** Result of Patient report outcome measures (PROM)

	HOOS-symptom	HOOS-pain	HOOS-ADL	HOOS-SR	HOOS-QOL	HOOS	
Pre-operative							
NMO	54.17 ± 20.87	32.71 ± 2.42	27.33 ± 17.39	15.63 ± 15.19	9.38 ± 11.46	29.06 ± 13.94	
Non-NMO	50.00 ± 21.52	40.63 ± 10.39	47.86 ± 15.20	26.82 ± 12.15	16.93 ± 17.04	41.12 ± 13;82	
* P* value	0.584	0.249	0.001	0.022	0.269	0.019	
Post-operative							
NMO	94.17 ± 9.96	95.83 ± 6.34	90.69 ± 8.42	71.35 ± 25.90	72.40 ± 26.04	81.70 ± 2.16	
Non-NMO	88.33 ± 10.39	92.92 ± 7.86	94.61 ± 7.74	84.64 ± 0.21	82.55 ± 13.16	82.79 ± 1.84	
*P* value	0.141	0.210	0.596	0.163	0.269	0.538	

	EQ-mobility	EQ-self care	EQ-usual activity	EQ-pain	EQ-anxiety	EQ-VAS	VAS
Pre-operative							
NMO	4.42 ± 0.67	3.71 ± 0.62	4.42 ± 0.67	4.08 ± 0.99	4.08 ± 0.99	30.58 ± 13.37	8.17 ± 2.08
Non-NMO	1.54 ± 0.17	1.19 ± 0.08	1.37 ± 0.14	1.42 ± 0.15	3.67 ± 0.48	49.79 ± 16.36	7.79 ± 1.69
* P* value	0.004	0.123	< 0.001	0.322	0.097	0.001	0.565
Post-operative							
NMO	1.92 ± 1.16	1.25 ± 0.45	2.08 ± 0.79	1.92 ± 1.08	1.67 ± 0.98	71.67 ± 16.97	1.25 ± 2.38
Non-NMO	1.46 ± 0.58	1.17 ± 0.38	1.38 ± 0.49	1.42 ± 0.50	1.21 ± 0.41	88.00 ± 9.30	1.00 ± 0.93
* P* value	0.496	0.871	0.014	0.088	0.071	0.035	0.698

### Radiographic outcome

**Fig. 3:**
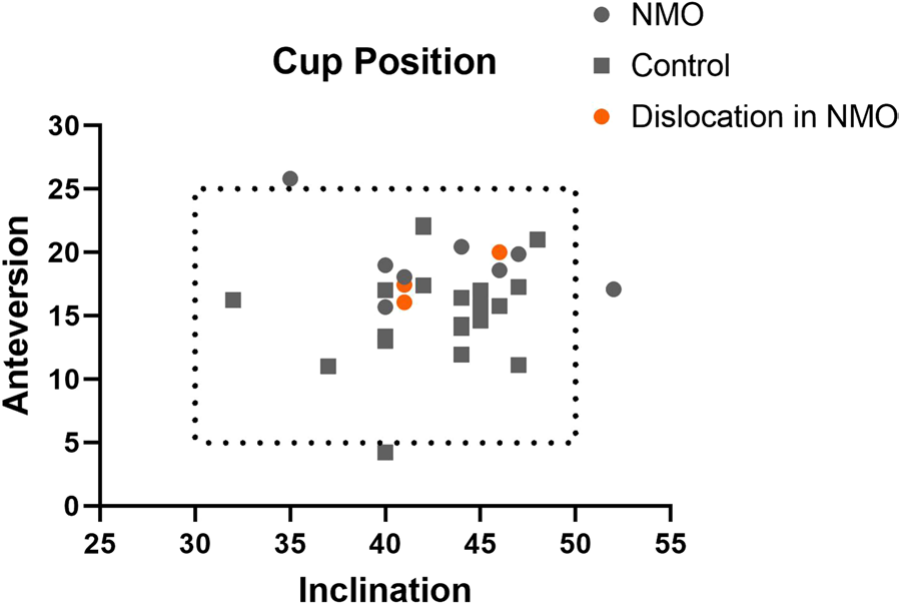
Cup position

One case in NMOSD group and two cases in non-NMOSD group had acetabular cup placed outside of the Lewinnek’s safe zone (Fig. 3). Three cases had limb length discrepancy(LLD) greater than 10 mm. There was no significant difference in terms of head size, LLD, and cup position between the two groups.(Table 4).

**Table 4:** Radiographic outcome

	NMOSD (n = 12)	Non-NMOSD (n = 24)	*P* value
Inclination (°)	43.17 ± 4.41	42.63 ± 3.88	0.708
Anteversion (°)	18.69 ± 2.76	15.22 ± 4.06	0.012
Cup outlier	2/12	1/24	0.253
LLD	6.09 ± 7.65	5.69 ± 4.93	0.849
LLD > 10 mm	2/12	1/24	0.253
Head size	32.33 ± 2.06	32.83 ± 2.88	0.596

^*^Data were displayed as means and standard deviation and rate

### Complications

The dislocation rate is significantly higher in the NMOSD group (*P* = 0.031). Three cases in the NMOSD had posterior dislocation in the first 3 months after surgery. Radiographic analysis showed that the cup position were within the Lewinnek’s safe zone in these three cases. No statistically significant difference was found in terms cup inclination and percentage of cups placed in Lewinnek’s safe zone. Cup anteversion was significantly higher in the NMOSD group (18.69° vs 15.22° *P* = 0.012). Two cases were treated with closed reduction and neither of them had recurrent dislocation until the latest follow-up. Another case is a 34 years old female who had dislocated her hip joint posteriorly 4 times during the last two years. To find out the cause for recurrent dislocation in this patient, CT with metal artificial reduction was conducted, which showed cup inclination of 41 degree and cup anteversion of 16 degree. Physical examination showed 4 + /5 in muscle strength and spasticity in bilateral lower limbs. No periprosthetic joint infection, periprosthetic fracture, aseptic loosening and other major complications were reported until the latest follow-up.

## Discussion

NMOSD was once thought of as a variant of multiple sclerosis (MS). However, researches have shown that NMOSD presents a more serious clinical condition than MS and is a different pathophysiological process which requires different treatment strategy [12, 13]. The prognosis of NMOSD are generally poor, with high levels of associated disability [5, 6]. The outcome of NMOSD patients undergoing THA have remained unknown. In this series, NMOSD patients who undergo THA are subject to an increased risk of dislocation. Comparing with those without the disease, similar improvement in functional outcome can be achieved with THA while the patient reported overall health status remained worse in patients with NMOSD.

NMOSD patients may become candidate for THA because of increased risk of trauma and osteonecrosis induced by steroids. Because of high prevalence of vision loss, limb weakness and spasticity, it has been reported that NMOSD patients had a significantly higher risk of falling and fracture [14]. The use of steroids are common among NMOSD patients, which may induce osteonecrosis. It is reported that nearly 30% of osteonecrosis of femoral head are steroids-induced in China [15]. In our series, 11 cases were diagnosed with steroids-induced osteonecrosis. Another patient had femoral neck fracture and received internal fixation initially. This patient later developed osteonecrosis and received THA.

To date, we found no literature reporting on the outcome following THA in NMOSD patients. However, it has been reported that patients with neuromuscular diseases including MS are subject to worse functional outcome and higher complication rate after total joint arthroplasty [16, 17]. In our series, NMOSD patients experienced more difficulties in activities in daily life, sports and recreation prior to surgery comparing with non-NMOSD patients. The average post-operative general HOOS score and HOOS score in symptom, pain, ADL, SR and QOL was similar in both groups. Considering the pre-operative HOOS score was significantly lower in NMOSD patients, significant improvement in functional outcome was achieved. No significant difference was found in VAS score both pre-operatively and post-operatively between the two groups. EQ-5D and EQ-VAS were used to assess the self-reported health status. NMOSD patients had significantly worse performance in EQ-usual activity. EQ-VAS was assessed on the scale between 1 to 100, with 100 means the best health patient feels and 0 means worst health patient feels. NMOSD patients had significantly lower EQ-VAS score prior to and after surgery. For NMOSD patients, THA provided significant improvement in functional outcome of the hip. However, the self-reported health score remained significantly lower in NMOSD group.

In our series, the rate of dislocation is 25% (3/12) in NMOSD patients, which is significantly higher than that in the control group. There was no significant difference in terms of BMI, head size, LLD, and cup inclination between the two groups. Three patients had posterior dislocation in the first three months, two of them had lower limb weakness and spasticity. Head size and cup position may be associated with risk of dislocation as well [18]. The head size was 32 mm in two patients and 28 mm in another one. Acetabular cup was placed in the Lewinnek’s safe zone in all three cases. However, the reliability of assessing cup position based on Lewinnek’s safe zone has been questioned [19]. New methods have been developed to assess safe zone more specifically with consideration of spinopelvic relationship and dynamic simulation of post-operative movement [20]. In our series, Lewinnek’s safe zone was not successful in predicting disability following THA. One patient in our series had 4 recurrent dislocations, she also suffered from two recurrent attacks of NMOSD and had progressive limb weakness and spasticity. Her muscle strength was rated as 4−/5 at the latest follow-up. Strict hip precautions was advised and the patient had not dislocated her hip again in the last 6 months. Neuromuscular disease including Parkinson’s disease and cerebral palsy are risk factors for dislocation following THA [18], which could explain the high dislocation rate in the NOMSD group after other factors have been analyzed. Considering that NMOSD patients are subject to recurrent attacks of myelitis and may develop progressive limb weakness and spasticity. hip precaution and rehabilitation should be patient-specific and time-specific.

This study has several limitations: 1. Limited number of cases was included and a single-center study design was applied. Therefore, this study is potentially underpowered and may cause bias. 2. The process of selecting control patients may cause bias although a matched case–control design was conducted to reduce bias caused by confounding factors. 3. The average follow-up time of this study was 24 months, long-term functional outcome and complication rate were not evaluated.

## Conclusion

THA is a viable option for the treatment of osteonecrosis in NMOSD patients. Satisfactory improvement in functional outcome was achieved. However, there is a significantly increased risk of dislocation after THA in the NMOSD group. Due to the natural process of NMOSD, rehabilitation and hip precaution should be patient-specific and time-specific.

## Data Availability

Raw data and materials of this study is deposited in the local server of Peking Union Medical College Hospital for safety and confidentiality purpose. Our data are available upon request for non-commercial research purpose.
